# PEM Electrochemical Hydrogen Compression with Sputtered Pt Catalysts

**DOI:** 10.3390/membranes13060594

**Published:** 2023-06-12

**Authors:** Galin Borisov, Nevelin Borisov, Jochen Heiss, Uwe Schnakenberg, Evelina Slavcheva

**Affiliations:** 1 “Acad. Evgeni Budevski” Institute of Electrochemistry and Energy Systems—Bulgarian Academy of Sciences, Acad. G. Bonchev Street 10, 1113 Sofia, Bulgaria; 2 Institute of Materials in Electrical Engineering I, RWTH, Aachen University, Sommerfeld Street 24, 52074 Aachen, Germany

**Keywords:** hydrogen, sputtered Pt, electrocatalysts, membrane electrode assemblies, electrochemical hydrogen pump

## Abstract

This work presents research on thin magnetron-sputtered platinum (Pt) films deposited over commercial gas diffusion electrodes and applied to convert and pressurize hydrogen in an electrochemical hydrogen pump. The electrodes were integrated into a membrane electrode assembly with a proton conductive membrane. Their electrocatalytic efficiency toward hydrogen oxidation and hydrogen evolution reactions was studied in a self-made laboratory test cell by means of steady-state polarization curves and cell voltage measurements (U/j and U/p_diff_ characteristics). The achieved current density at a cell voltage of 0.5 V, the atmospheric pressure of the input hydrogen, and a temperature of 60 °C was more than 1.3 A cm^−2^. The registered increase in the cell voltage with the increasing pressure was only 0.05 mV bar^−1^. Comparative data with commercial E-TEK electrodes reveal the superior catalyst performance and essential cost reduction of the electrochemical hydrogen conversion on the sputtered Pt films.

## 1. Introduction

Hydrogen is a flexible energy carrier that is considered to be a clean sustainable alternative to fossil fuels. It is capable of addressing both environmental and technical issues, such as zero carbon footprint, grid balancing, and the energy autonomy of remote consumers [1,2,3]. One of the most technically challenging barriers in regard to the broad industrial deployment of hydrogen energetics is the development of a safe, compact, and cost-effective hydrogen storage technology [4]. Hydrogen can be stored in a gaseous or liquid form, in metals, glass microspheres, carbon nanotubes, etc. Its efficient storage requires the pressurization of hydrogen to a medium or high pressure [5]. Electrochemical hydrogen compression (EHC) is a highly efficient, low-maintenance, and silent technology, which is considered to be a potentially viable and cost-competitive alternative to traditionally used mechanical piston compression [6,7,8]. These compressors, also called hydrogen pumps, offer advantages such as compact cell/stack design; a lack of moving parts and, therefore, a lack of wear and tear problems; and the possibility to operate without permanent service [9]. The efficiency of EHC exceeds 80%, and the output pressure per stage at low power can reach more than 40–50 bars [10]. Moreover, this type of hydrogen energy conversion is fully compatible both with the high purity hydrogen (>99.9999%) produced via water electrolysis and the hydrogen generated by reforming mixed with CO-based impurities [11].

The H_2_/H_2_ cell based on low-temperature proton exchange membranes was originally invented in the 1960s [12,13]. Furthermore, it has been developed and broadly used for various processes, such as electrochemical hydrogen pumping and purification in FC systems [14], the extraction of hydrogen from hydrogen-containing gases [15], steam electrolysis to produce pure hydrogen [16], the dehydration of methane gas for coupling reactions [17], and sensor applications [18].

**Figure 1:**
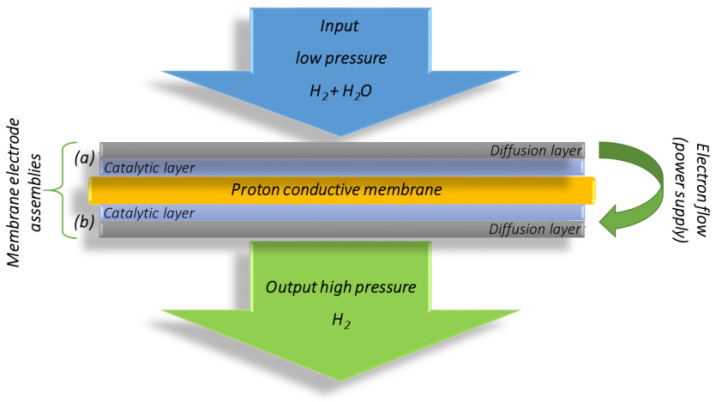
Schematic presentation of the electrochemical hydrogen compressor/pump working principle in a single stage mode: (**a**) anode; (**b**) cathode.

In this study, the working principle of the H_2_/H_2_ cell (Figure 1) was adapted for the electrochemical characterization of polymer exchange membrane electrochemical hydrogen compression (PEM EHC) electrodes and the investigation of the EHC process under various test conditions.

The key active element where electrochemical energy conversion takes place is the membrane electrode assembly (MEA), consisting of two gas diffusion electrodes (GDE) separated by a polymer proton conductive membrane. The anode compartment of the EHC cell is fed with wet hydrogen gas at a low input pressure. The electrochemical oxidation of hydrogen (HOR) on the anode proceeds according to partial Reaction (1):

H_2_ (p_an_) → 2H^+^ + 2e (1)

The obtained protons move through the polymer membrane and are reduced on the cathode (HER) according to partial Reaction (2):

2H^+^ + 2e^−^ → H_2_ (p_cat_) (2)

The overall electrochemical hydrogen conversion proceeds according to (3):

H_2_ (p_an_) → H_2_ (p_cat_) (3)

The produced hydrogen is accumulated in the cathode chamber, where the pressure increases gradually.

Although the partial electrode reactions are well known and have been broadly investigated, there are many factors that influence the performance of the electrochemical hydrogen pump based on PEM technology such as the hydrogen crossover, the water transport through the membrane, the MEA architecture, the proton conductivity of the membrane, the porosity and hydrophobicity of the gas diffusion electrodes, the activity and durability of the catalysts, etc. The state-of-the-art proton conductive membrane is the Nafion 117 membrane, known for its high conductivity, mechanical stability, and well-determined operating temperature range [19]. The gas diffusion EHC electrodes need to possess a highly developed surface, ensuring efficient catalyst utilization, optimized porosity, and hydrophobicity to facilitate the transport of the reagents and to prevent the flooding of the electrodes. The commercially available gas diffusion electrodes (GDE) for PEM FC fulfill these requirements and can be successfully applied to EHC [20]. The catalysts used for both of the partial electrode reactions are usually noble metals—platinum (Pt), palladium (Pd), or their alloys [21]. The active metal is dispersed on a supporting material with a highly developed surface and excellent electric conductivity (carbon blacks, nanoparticles, fibers, or nanotubes). The metallic part of the catalyst varies in the range of 20–40 wt.%, while the catalytic loading exceeds 1–2 mg_Me_·cm^−2^ at the anode and often reaches more than 2–3 mg_Me_·cm^−2^ at the cathode [22]. The active metal can be spread on the surface of the gas diffusion layer through a variety of methods among which the methods that are most often used are the “doctor Blade” technique or the spraying of catalytic ink [23].

In the last decade, the physical vapor deposition (PVD) method of magnetron sputtering, broadly used in microelectronics, has received renewed interest for the deposition of catalysts, particularly for fuel and electrolysis cells with a polymer electrolyte membrane [24,25,26,27]. It has been introduced as an alternative to the classical wet-chemical, sol–gel, and thermal decomposition methods, which produce catalytic powders on carbon substrates with a highly developed surface. In contrast, the catalysts prepared by magnetron sputtering are deposited as thin compact films with a precisely controlled surface structure and ultralow loadings (down to 10 μm cm^−2^) [28]. Sputtered Pt, iridium (Ir), their alloys, oxides, and various multilayer combinations have shown remarkable mechanical stability combined with high catalytic efficiency toward the partial electrode reactions. These films are already utilized in fuel and water electrolysis cells as well as in the reversible unitized hydrogen energy conversion systems.

In this work, we investigated the catalytic efficiency of thin magnetron-sputtered Pt films toward the partial electrode reactions in a custom-made electrochemical hydrogen compressor/pump to verify the applicability of the method and to compare the cost efficiency of the sputtered platinum films in regard to the classical carbon-supported Pt.

## 2. Experimental

The catalytic films under study were deposited on commercial H2315C2 gas diffusion layers (GDLs) provided by Freudenberg (Weinheim, Germany). The GDL with a thickness of 250 μm had an upper microporous layer (MPL) consisting of graphite nanoparticles mixed with polytetrafluorethylene (PTFE). MPL facilitated the transport of the reactants to and from the electrode active sites and ensured an even distribution of both the reactant and the current density over the electrode surface. Pt films were magnetron-sputtered over the microporous layer. The metal loading on each electrode was 0.25 mg_Pt_ cm^−2^. The detailed description of the Pt layer deposition process was reported elsewhere [28].

**Figure 2:**
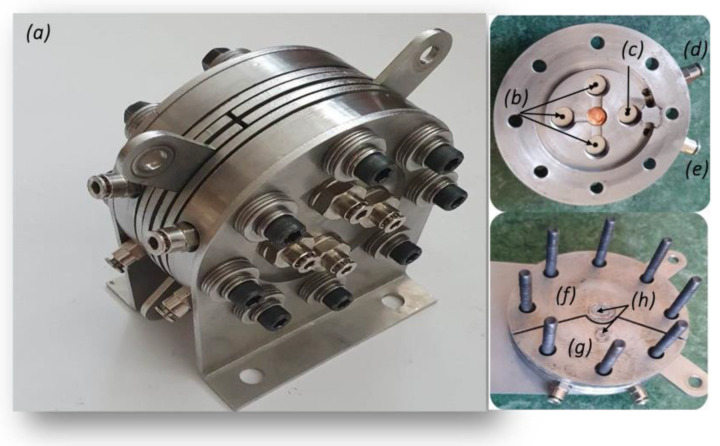
Picture of the self-designed electrochemical hydrogen compression cell: (**a**) main view, (**b**) input and output hydrogen flow fields, (**c**) hydrogen flow field for reference electrode, (**d**,**e**) fittings for temperature regulation, (**f**) main current collector, (**g**) reference electrode electrical contact, and (**h**) titanium foam for gas distribution.

Two Pt-coated GDEs with area of 1 cm^2^ were attached to the coated sides on both sides of a Nafion^®^ 117 polymer electrolyte membrane, applying mechanical force of 20 Nm, and were tested in a custom-made EHC cell, which is presented in Figure 2.

The cell was equipped with reference electrodes (Pt/XC72 ETEK, 40 % wt. Pt) both in the anode and the cathode compartments, allowing us to simultaneously study the polarization of each of the electrodes and also to follow the change in the cell voltage in order to record the U_cell_/j and U_cell_/p_diff_ characteristics.

The hydrogen gas feeding the EHC was generated in a laboratory PEM electrolyzer and was delivered at atmospheric pressure (0 bar overpressure) directly to the anode compartment of the EHC cell. The catalytic efficiency of the sputtered Pt films toward the HOR and HER was investigated through linear sweep voltammetry (polarization curves and cell voltage measurements) at varying temperatures in a range of up to 60 °C at differential pressures of up to 2.5 bar. The performance of the MEA under study was compared with that of MEA with state-of-the-art commercial electrodes (E-TEK) with a platinum loading of 1 mg_Pt_ cm^−2^. All experiments were carried out using Gamry 1010E Potentiostat/Galvanostat (Gamry Instruments Inc., Warminster, PA, USA).

## 3. Results and Discussion

It has been reported previously that the parameters of the sputtering process (the temperature, partial pressure of the inert and reactive gases, dc power, etc.) have a decisive effect on the properties of the deposited films, such as density, surface morphology, porosity, and mechanical stability. In [28,29], it was shown that, by varying the sputtering parameters, it is possible to essentially modify the film morphology and, thus, to tailor its electrochemical behavior. High sputtering pressures are beneficial, ensuring a highly developed active surface and increased catalytic activity. The samples deposited at 9 Pa proved to be better catalysts compared to commercial carbon-supported Pt regarding both the oxygen reduction reaction and catalyst utilization [28]. The deposition regime with a 100 W sputtering power and a 9 Pa argon pressure has been recommended as an optimal one for the deposition of low-Pt-loaded catalytic films on gas diffusion substrates, with a potential application as highly efficient cathodes in low-power PEMFC [30].

**Figure 3:**
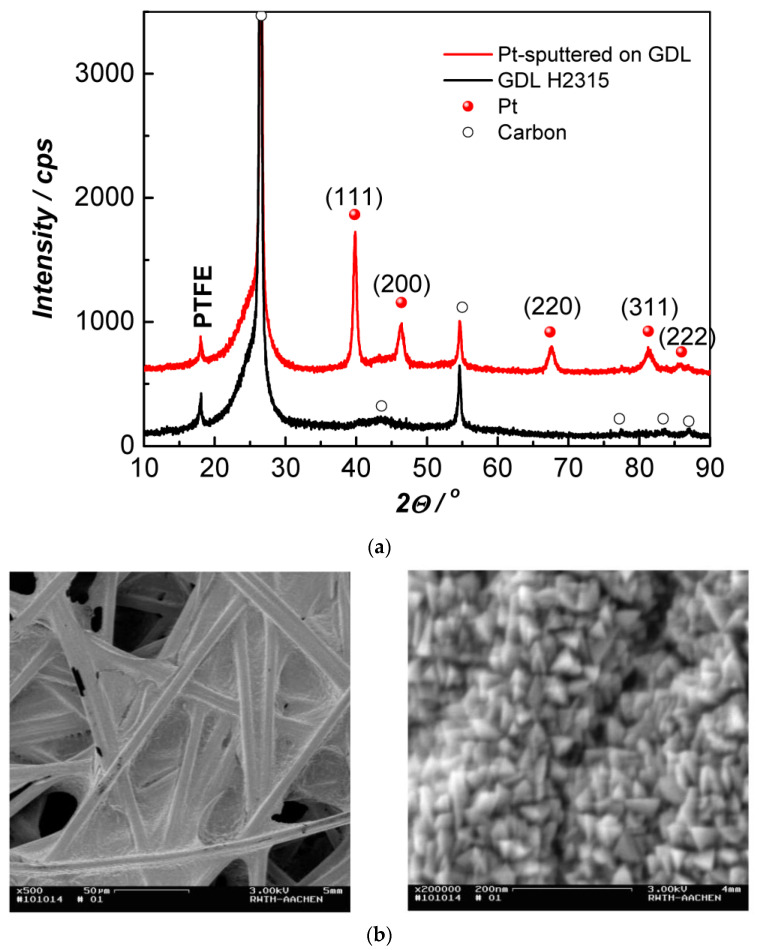
(**a**) XRD data of bare (as delivered) gas diffusion layer with integrated MPL (black line) and of GDL covered with dc magnetron-sputtered platinum film (red line) and SEM images (**b**) of the sputtered Pt film at two different magnifications.

The platinum films investigated herein were deposited at the optimal sputtering parameters (a dc power of 100 W at an argon gas flow rate of 100 sccm, a pressure of 9 Pa, and a distance between the Pt target and the substrate of 78 mm), ensuring the polycrystalline structure of Pt with the predominant (111) orientation and optimal porosity. Their crystal structure and phase identification were analyzed through X-ray diffraction (XRD) and scanning electron microscopy (SEM). The obtained results are presented in Figure 3a,b.

The diffractograms show the peaks of carbon and polytetrafluoroethylene (PTFE) as well as the peaks typical for polycrystalline Pt. The sizes of the Pt crystallites (15 ± 1 nm) and the Pt cell parameter (3.9205 Å) were calculated using the (111) peak, which is the most intensive one.

On the SEM images presented in Figure 3b, it can be seen that the platinum particles are connected, forming a continuous microporous film with a highly developed surface that follows the texture of the substrate.

**Figure 4:**
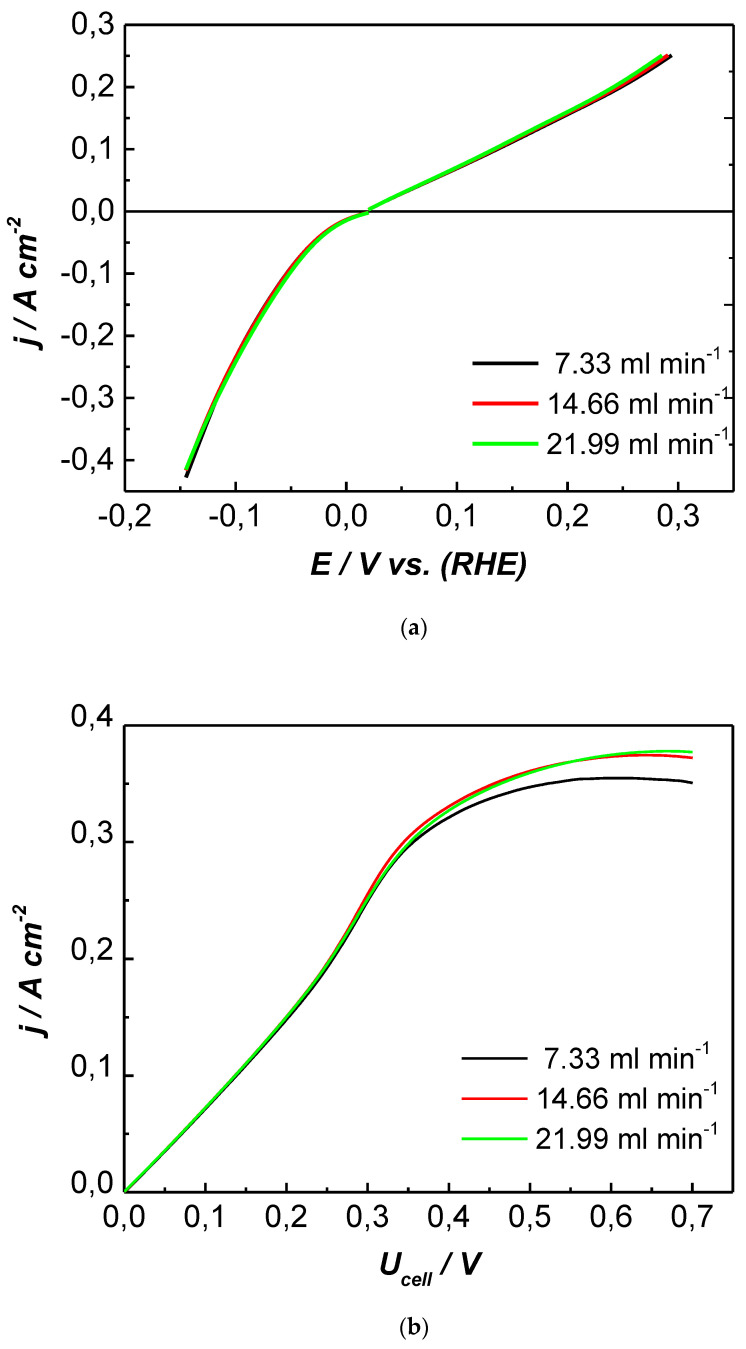
Polarization curves (**a**) and voltampere characteristics (**b**) of electrochemical hydrogen conversion on MEA with sputtered Pt catalysts at room temperature and varying hydrogen flow rates, p_an_ = p_cat_ = 0 bar, and potential scan rate of 1 mV s^−1^.

The catalytic efficiency of the sputtered Pt was firstly investigated in an “open” EHC cell, i.e., at atmospheric input and output pressures (p_an_ = p_cat_ = 0 bar). In Figure 4a,b, the anodic and cathodic polarization curves (HOR and HER, respectively) and the voltampere characteristics (the j/U_cell_ curves) recorded at three different values of the input hydrogen flow rate, f_H2_, at room temperature are shown.

It can be seen that, at low overpotentials, the change in the hydrogen flow rate had no influence on either HOR or HER (Figure 4a). The linear dependence at low current densities indicates the predominant ohmic character of polarization. At the same time, the voltampere characteristics in Figure 4b showed a linear j/U_cell_ dependence of up to 0.25 V, while, at higher voltages, the curves deviated from linearity. In this region, the process slowed down due to transport limitations. Under these conditions, the current density was affected by the hydrogen flow rate. It slightly increased with the increased f_H2_ as more hydrogen penetrated the gas diffusion layer and reached the triple-phase boundary reactive gas (H_2_)/electrolyte/catalyst, where the electrochemical reaction took place. At a cell voltage of 0.7 V, the increase in f_H2_ from 7.33 to 21.99 mL min^−1^ resulted in a higher current density of about 0.03 A cm^−2^.

**Figure 5:**
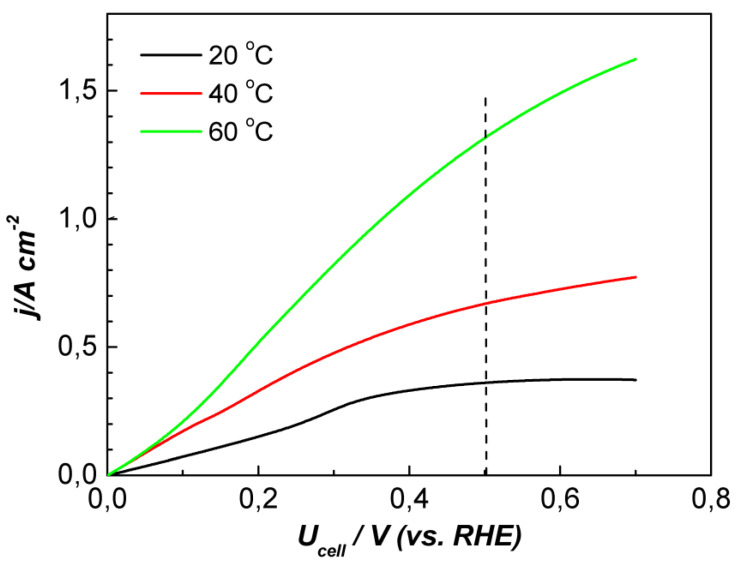
Voltampere characteristics of electrochemical hydrogen conversion on MEA with sputtered Pt catalyst at varying temperatures, f_H2_ = 7.33 mL min^−1^, p_an_ = p_cat_ = 0 bar, and potential scan rate of 1 mV s^−1^.

The influence of temperature on the intensity of electrochemical hydrogen conversion is demonstrated in Figure 5. As expected, the higher the temperature, the more intensive the reaction is. The current density at 0.5 V increased from 0.36 A cm^−2^ at 20 °C and to 1.3 A cm^−2^ at 60 °C. These results indicate a decrease in the activation energy of the process. At the same time, the region of the curve where the transport limitations appeared shifted to higher voltages with increasing temperature.

In order to investigate the effect of differential pressure, p_diff_, on the efficiency of EHC, the cell was closed hermetically. The input pressure, p_an_, and hydrogen flow into the anodic chamber were kept constant. The pressure in the cathodic chamber, p_cat_, depended on the rate of hydrogen conversion; on the current density, j; and on the test duration, respectively. The higher the applied current density, the faster p_cat_ and, thus, the differential pressure between both cell chambers increased (p_diff_ = p_cat_ − p_an_).

**Figure 6:**
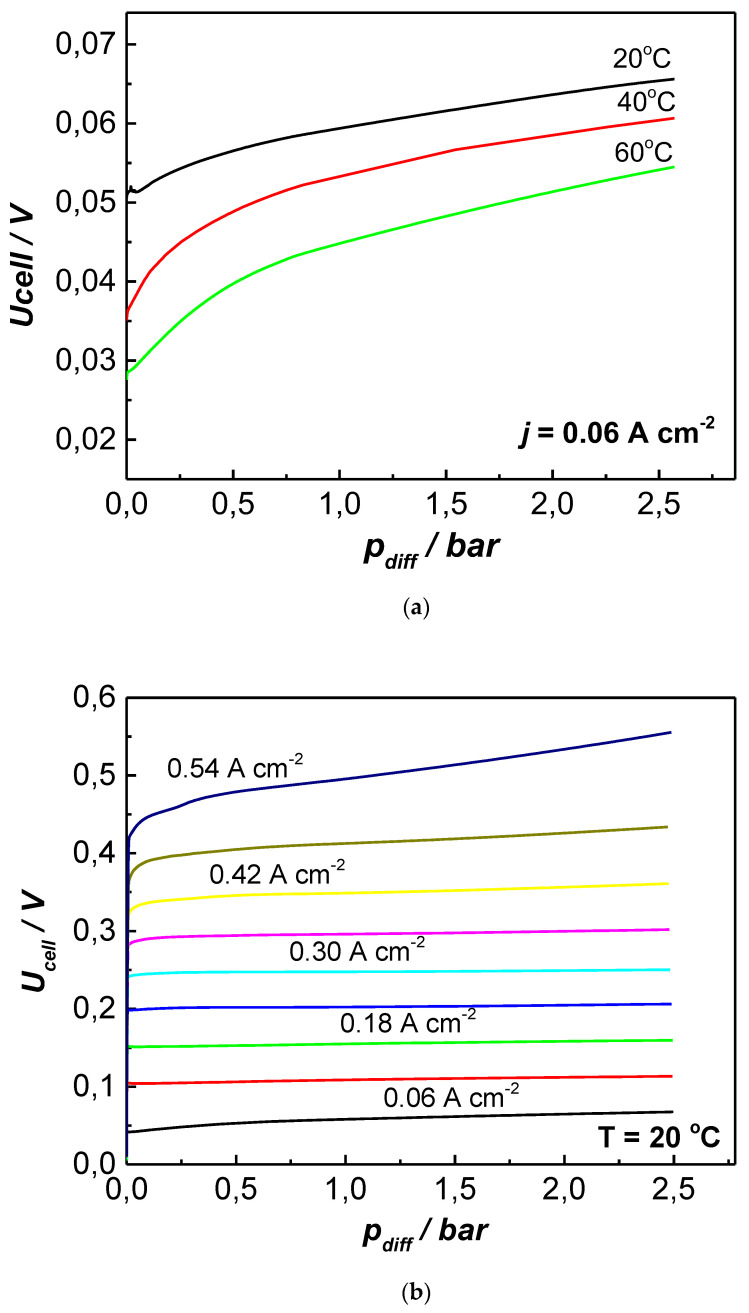
Influence of differential pressure on EHC cell performance at varying temperatures (**a**) and varying current densities, with step of 0.06 A cm^−2^ (**b**); f_H2_ = 7.33 mL min^−1^; potential scan rate of 1 mV s^−1^.

Figure 6 presents the influence of temperature (Figure 6a) and the applied current density (Figure 6b) on U_cell_/p_diff_ dependence. The figures reveal that the positive effect of temperature on the process’s efficiency, registered in the open EHC cell (Figure 5), was sustained at a higher differential pressure (Figure 6a) and that the cell voltage generally increased with increasing pressure. The latter effect was more pronounced (the slope of the U_cell_/p_diff_ curve was steeper) at the initial increase in p_diff_ in the range 0–1.0 bar.

**Figure 7:**
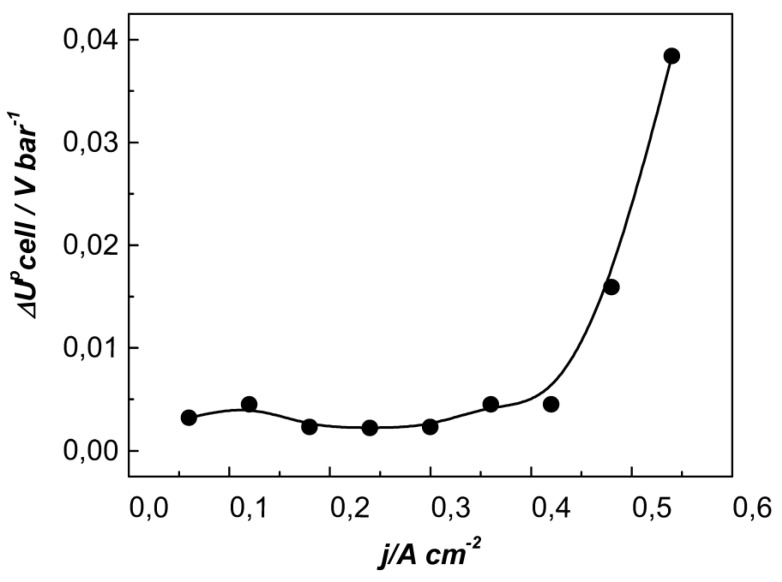
The change in EHC voltage normalized to p_diff_ with the increasing current density.

In the current density range of up to 0.4 A cm^−2^, the cell performance followed a similar trend in the pressure range of 0–1.5 bar (e.g., U_ce_ increases with p_diff_), while, at a higher pressure, U_cel_ stabilized and did not change essentially till test termination at 2.5 bar. The further increase in the applied current density resulted in a more pronounced initial increase in cell voltage and a steeper slope of the curves without the plateau observed at a moderate p_diff_. This effect is well demonstrated in Figure 7, where the change in U_cell_ is normalized to the p_diff_ (ΔU^p^_cell_, mV bar^−1^) and is presented as a function of the current density.

**Figure 8:**
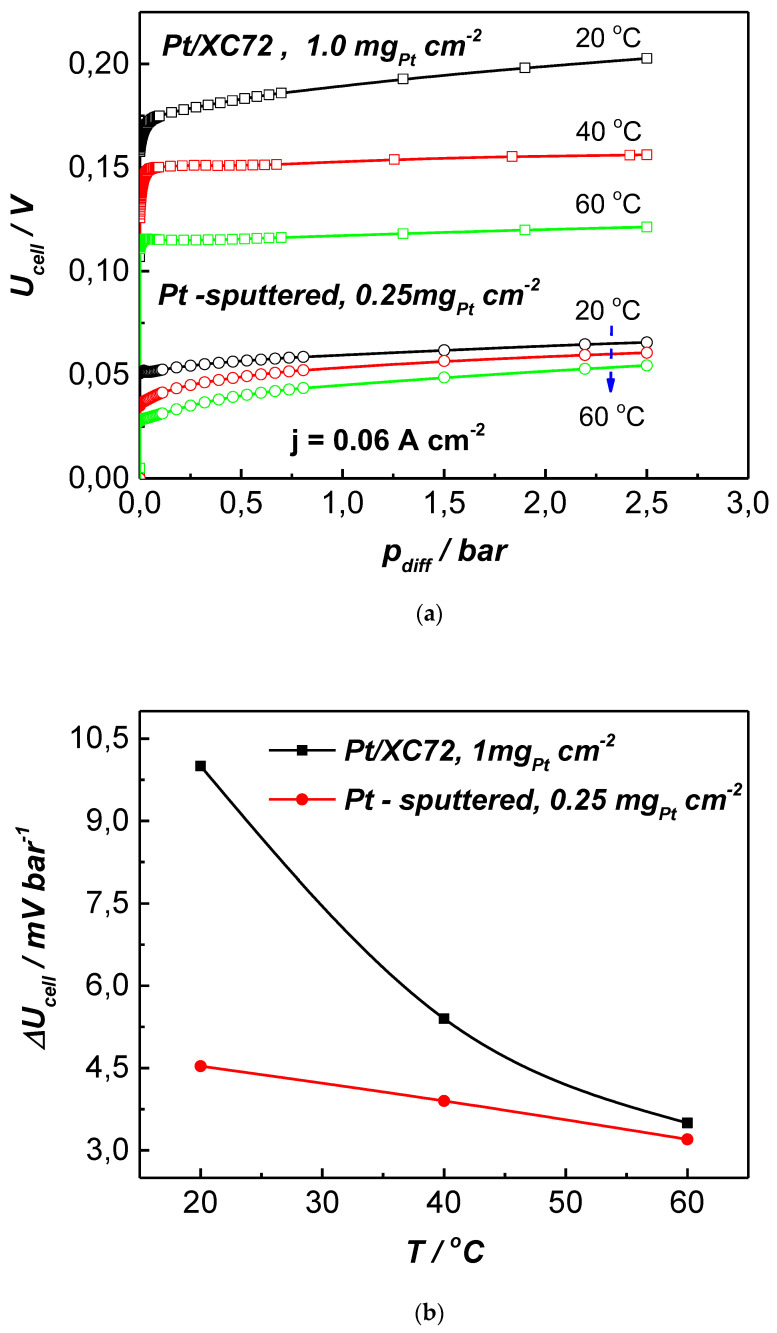
Influence of the type of platinum catalyst on the EHC cell performance at constant current density of 0.06 A cm^−2^: (**a**) change of U_cell_ with differential presure_l_; (**b**) change of ΔU_cell_ with temperature.

In the current study, we performed comparative EHC tests at identical conditions on the MEA with commercial gas diffusion electrodes containing catalytic platinum nanoparticles dispersed on carbon black (Pt/XC72). Figure 8 presents the obtained experimental data illustrating the influence of differential pressure on the cell performance at varying temperatures and the change in U_cell_ normalized to the p_diff_ (ΔU^p^_cell_) as a function of temperature.

It can be seen that, in the whole temperature range tested, the gas diffusion electrodes with magnetron-sputtered Pt showed better efficiency in comparison with those containing Pt/XC72 at four times higher noble metal loading (0.25 mg_Pt_ cm^−2^ vs. 1.0 mg_Pt_ cm^−2^). The U_cell_/p_diff_ dependence of both MEAs followed the same trend, but the cell voltage was about 0.125 mV higher for the Pt/XC72 catalyst. Accordingly, ΔU^p^_cell_/T decreased with increasing temperature for both MEAs, while the calculated drop of the normalized voltage was lower for the MEA with the sputtered Pt catalysts, indicating the thermal stability of the cell performance. The observed difference in the effect of temperature could be prescribed to the different thicknesses of both catalytic layers and the different ohmic resistances of the electrodes. The Pt/sputter electrode was about 250 μm thick vs. the nearly double 400 μm thickness of the commercial ETEK electrode. Moreover, using the sputter technology, the catalyst dispersed directly on the diffusion substrate penetrated into the depth of the GDL, which improved the electronic conductivity. In the case of the commercial electrode, additional contact resistance may occur between the carbon-supported Pt nanoparticles decorating its thermal behavior.

The superior performance of the sputtered Pt films could be explained by the 3D architecture of the catalytic layer. During sputter deposition, the platinum atoms followed the texture of the porous substrate and penetrated the GDL, forming a continuous catalytic film in its depth and thus ensuring a highly developed active surface area for the electrochemical reactions. The result of this was the better utilization of the catalyst and enhanced MEA performance.

The results obtained demonstrate the effectiveness of the sputtered platinum films as catalysts for the HOR and HER in the electrochemical hydrogen compression cells. They are in good accordance with our previously reported data obtained in the EasyTest cell, proving the superior catalytic activity of the sputtered Pt films compared to the carbon-supported Pt toward the partial electrode reactions in hydrogen fuel cells and water electrolyzers at low and moderate operating regimes and the worse performance at high currents due to the transport limitations of the proceeding reactions [28,29].

## 4. Conclusions

The developed laboratory test cell can operate as a hydrogen pump and electrochemical compressor. The design proposed, with two integrated reference electrodes, allows for simultaneously studying both partial electrode reactions and the MEA performance in a broad range of operating temperatures and differential pressures. The investigated thin magnetron-sputtered platinum films proved to be efficient catalysts for electrochemical hydrogen conversion. The rate of the reaction reached a current density of 1.3 A cm^−2^ at a cell voltage of 0.5 V and at 60 °C. In comparison to the commercial carbon-supported platinum, the sputtered films demonstrated superior cost efficiency combined with a lower inclination to performance degradation with an increase in the cell’s differential pressure.

## Data Availability

Not applicable.
